# Primary angiitis of the central nervous system: predictors of stroke during immunosuppressant treatment

**DOI:** 10.1186/s13244-026-02217-4

**Published:** 2026-02-17

**Authors:** Franca Wagner, Jakob Heimer, Pasquale Mordasini, Thomas Hundsberger, Roman Guggenberger, Anna L. Falkowski, Simon Wildermuth, Sebastian Leschka, Tobias Johannes Dietrich, Tim Steffen Fischer

**Affiliations:** 1 Institute of Diagnostic and Interventional Neuroradiology, Center for Imaging and Minimally Invasive Therapies, Kantonsspital Aarau (KSA), Aarau, Switzerland; 2 University Institute of Neuroradiology, Bern University Hospital, University of Bern, Bern, Switzerland; 3 Clinical Trials Unit, HOCH, Cantonal Hospital St. Gallen, St. Gallen, Switzerland; 4 Division of Radiology and Nuclear Medicine, HOCH, Cantonal Hospital St. Gallen, St. Gallen, Switzerland; 5 Department of Neurology and Oncology, HOCH, Cantonal Hospital St. Gallen, St. Gallen, Switzerland; 6 Clinic for Radiology and Nuclear Medicine, Cantonal Hospital Winterthur, Winterthur, Switzerland; 7 Faculty of Medicine, University of Zurich, Zurich, Switzerland

**Keywords:** Magnetic resonance imaging, Brain, Vasculitis, Contrast media

## Abstract

**Objective:**

To evaluate predictors of ischemic stroke in patients with primary angiitis of the central nervous system after initiation of immunosuppressive therapy.

**Materials and methods:**

This retrospective study included 204 MRI examinations of 23 patients with primary angiitis of the central nervous system, treated with immunosuppressive therapy between 2015 and 2020 at the University Hospital Bern and the Cantonal Hospital St. Gallen, Switzerland. Two senior neuroradiologists evaluated the MRI exams with regard to the occurrence and location of ischemic stroke and hemorrhage, as well as the following characteristics of inflamed vessels on 3D time-of-flight angiography and T1 dark-blood post contrast: signal intensity of vessel walls, length of enhancement, circular extent of enhancement, and stenosis. After matching ischemic strokes to their corresponding vessel, the temporal relationship of vessel alterations in accordance with therapy initiation and stroke onset was calculated.

**Results:**

The majority (77.6%) of observed strokes were in the vascular territory of an inflamed vessel. A significant, non-linear temporal relationship between the timing of MRI and the initiation of immunosuppression was found. The highest predicted probability of ischemic stroke was observed between 10 and 20 days after the initiation of immunosuppressant therapy, reaching approximately 12%. Out of all evaluated vessel characteristics, a higher degree of stenosis (Estimate: 0.93, *p* = 0.006) and a higher circularity of enhancement (Estimate: 0.76, *p* = 0.01) were significantly associated with a higher likelihood of stroke.

**Conclusions:**

A better understanding of unfavorable constellations (critical timeframe, characteristic vessel wall changes) in patients treated for primary angiitis of the central nervous system may help to prevent secondary ischemic strokes.

**Critical relevance statement:**

A better understanding of ischemic stroke predictors in patients treated for primary angiitis of the central nervous system may prompt closer monitoring or therapy adjustment.

**Key Points:**

- To evaluate risk factors for ischemic stroke in patients treated for primary angiitis of the central nervous system.
- Higher degree of stenosis and circular enhancement are associated with a higher likelihood of ischemic strokes, which typically occur between 10 and 20 days after therapy onset.
- Data obtained from this may prompt closer monitoring or therapy adjustment.

**Graphical Abstract:**

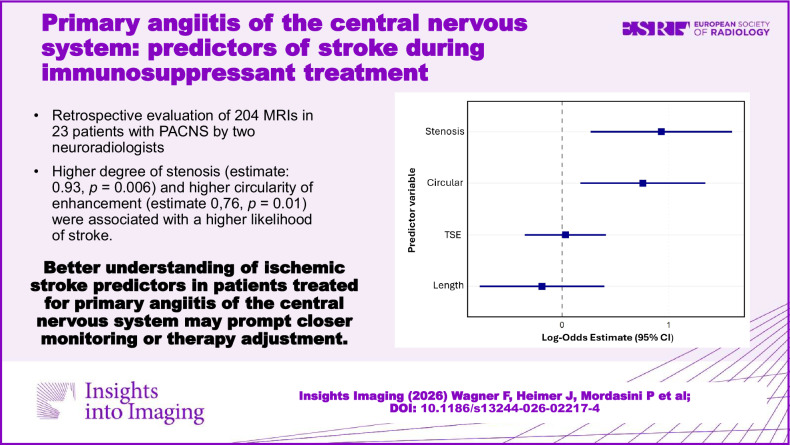

## Introduction

Primary angiitis of the central nervous system (PACNS) is a rare and severe subtype of vasculitis, with inflammatory involvement limited to the brain, spinal cord, and leptomeninges [1, 2]. It is one of only few vasculitis subtypes limited to one organ only according to the revised Chapel Hill classification [3]. PACNS can be divided into large vessel (LV) vasculitis with involvement of large and medium-sized vessels and small vessel (SV) vasculitis, affecting small-sized vessels [4].

Patient symptoms related to PACNS are nonspecific and include headache, focal neurological deficits varying degrees of severity and altered cognition [1, 2, 5]. Typical manifestations include ischemia and, less frequently, hemorrhage [6]. The estimated prevalence in North America is 2.4/1,000,000 person-years with a male-to-female ratio of 1:1 [7] with a median age of 50 years [8].

Diagnosis of PACNS is challenging and based on three cornerstones: neuropathological, neuroimaging and clinical, including cerebrospinal fluid (CSF) analysis.

Neuropathologically, PACNS is characterized by granulomatous inflammation, lymphocytic cellular infiltrates and necrotizing vasculitis [6, 9]. Nonspecific findings include gliosis, perivascular mononuclear inflammation or infarction [9]. The sensitivity lies between 53 and 63% [10].

Digital Substation Angiography (DSA) may reveal vascular irregularities in LV-PACNS, but is, in contrast, usually unremarkable in patients with SV-PACNS [11]. The sensitivity has been reported as 40–90%, with a very low specificity of only 30% [12]. Conventional MRI sequences are usually abnormal in patients with PACNS but lack specificity. Typically, infarction or hemorrhagic lesions of different ages are noted [13]. MR 3D-Time-of-Flight Angiography 3D-(TOF-MRA) reveals stenoses of large and medium-sized intracranial vessels and specific black-blood vessel wall imaging is used to detect inflammatory changes within the vessel wall [14–21]. SV-PACNS is only defined by neuropathology [4].

In adults with clinical suspicion of PACNS, CSF examination is recommended, especially to rule out differential diagnosis, e.g., post-infectious vasculitis [4]. CSF analyses are abnormal in 66–90% of patients with CNS vasculitis, showing lymphomonocytic pleocytosis or elevated protein levels [2, 5, 12]. Normal CSF analysis cannot exclude the diagnosis of PACNS [4].

The aim of this study was to evaluate the predictors for recurrent strokes in patients with PACNS under immunosuppressive therapy. Patients at risk can be identified and closely monitored. In the future, treatment modifications may even lead to the prevention of recurrent strokes.

## Materials and methods

This is a follow-up study of the previously published patient cohort of the Bern University Hospital and the St. Gallen Cantonal Hospital [17].

### Patient selection

Institutional Review Board (Ethics Committee of Eastern Switzerland, reference number 2021-02402) approval and written informed consent to participate were obtained from all patients. We retrospectively searched the databases of the University Hospital Bern and the Cantonal Hospital St. Gallen, Switzerland, to identify patients diagnosed with PACNS between 2015 and 2020. The following inclusion criteria were applied: All patients had to be diagnosed with PACNS by the Department of Neurology following an interdisciplinary discussion by the neurovascular board; besides imaging, including vessel wall imaging, a lumbar puncture was required. Biopsy and DSA were not mandatory for inclusion and were performed based on the board’s recommendation in less clear cases only. Immunosuppressant therapy had to be administered for at least 6 weeks; a short course of high-dose corticosteroids was not sufficient for inclusion into this study.

### Imaging

Imaging was performed at the University Hospital Bern or at the Cantonal Hospital St. Gallen. Most scanners were Siemens Healthineers, Erlangen, Germany; a few scanners were provided by Philips Health Systems, Gland, Switzerland. The following 3-T scanners were used: Siemens Magnetom Skyra, Skyra fit, Verio, Vida, Prisma fit and Trio. The following 1.5-T scanners were used: Siemens Magnetom Area, Avanto, Avanto fit, Symphony and Philips Intera. MRI protocols slightly varied due to the multicentricity of our study but uniformly consisted of DWI with a 0 and 1000 b-value for calculation of the apparent diffusion coefficient (ADC) and an axial T2-weighted sequence with or without fluid attenuation for CSF suppression. Additionally, TOF-MRA and T1-weighted, blood-suppressed dark blood sequence was used to evaluate vessel wall enhancement, either as turbo spin echo (TSE) or sampling perfection with application of optimized contrast using different flip-angle evolution (SPACE) on 3 T scanners only. At both sites, the post-contrast scans were performed after 1 min of contrast injection (0.1 mmol/kilogram body weight Gadobutrol; Gadovist, Bayer, Germany).

### Imaging evaluation

All MRIs were evaluated by two senior national board-certified neuroradiologists (except explicitly mentioned), one with an additional certification by the European Society of Neuroradiology. Both readers were blinded for clinical evaluation. The reading was performed in a randomized order (T.F. and F.W.).

Specific locations within the brain parenchyma were recorded by Reader 1 (T.F.) as described by the Alberta Stroke Program early CT score (ASPECTS) and posterior circulation acute stroke prognosis early CT score (pc-ASPECTS), with possible locations M1 to M6, C, IC, L and I (anterior circulation) and T, OL, M, P, C (posterior circulation). The following imaging findings were analyzed:

- Presence of infarction, visible on b1000 DWI, with a corresponding decrease in the ADC value.
- Presence of parenchymal hemorrhage. In addition to the location of hemorrhage, for graduation, the hemorrhagic transformation (HT) score, proposed by the European Cooperative Acute Stroke Study (ECASS) 2 [22], was used:
  - Hemorrhagic infarction 1 (HI-1), small petechial bleedings, absence of mass effect.
  - Hemorrhagic infarction 2 (HI-2), confluent petechial bleeding, absence of mass effect.
  - Parenchymal hemorrhage 1 (PH-1), Hematoma < 30% of the infarcted area, mild mass effect.
  - Parenchymal hemorrhage 2 (PH-2), Hematoma > 30% of the infarcted area, definite mass effect.

Each intracranial artery (internal carotid, anterior cerebral, middle cerebral, posterior cerebral, basilar and vertebral) was individually evaluated for signs of inflammation, including side.

The following location-dependent variables were measured:

- Signal intensity (SI) in gray-scale units of the vessel wall of an affected vessel on TSE or SPACE dark blood sequence after contrast.
- Length of vessel wall enhancement on TSE or SPACE dark blood sequence after contrast (millimeter, mm).
- Circular extent of the enhancement of the vessel wall on TSE or SPACE dark blood sequence after contrast (0–360°).
- Extent of the stenosis on 3D-TOF (0–100%).

In contrast to computed tomography examinations, where Hounsfield units are generally considered comparable, SI measurements on MRI may depend on software and hardware settings. To make the SI measurements taken from the TSE and SPACE dark blood sequences comparable, the “noise”-signal (SI of air) was evaluated by Reader 1 (T.F.). To normalize the SI values, the signal-to-noise ratio (SNR) was calculated and subsequently used. For measurements of the length of the vessel wall in case of a sloping vessel, multiple points were used to exactly follow the course of the vessel on 3D dark blood sequence with multiplane reconstruction. If only a conventional TSE was available, the axial plane was referenced with the TOF sequence to measure the vessel as precisely as possible. Circumferential extent of vessel wall enhancement was measured in the 3D dark blood sequence, on multi-planar reconstruction perpendicular to the long axis of the vessel. In case of a conventional TSE dark blood sequence, circumferential enhancement was estimated by evaluating enhancement of the inferior, anterior, posterior and superior vessel wall, each part of the vessel wall reflecting 90°. An example measurement is given in Supplementary Fig. S1.

Patient’s demographics and initial clinical presentation, especially the number of days since the patient was under treatment, were documented.

### Statistical methods

Statistical analysis was conducted with R (Version 4.4.1), and a *p*-value less than 0.05 was considered significant.

Baseline characteristics of the patient cohort and imaging assessments were summarized using descriptive statistics. Continuous data were reported as means and standard deviations (SD), while categorical data were reported as counts and percentages.

The extent of agreement between the measurements taken by the two readers was evaluated for each of the five variables, using the intraclass correlation coefficient (ICC) and Cohen’s κ for continuous and categorical variables. Measurements were treated as independent.

To evaluate predictors of vessel enhancement corresponding to an acute infarction, a generalized linear mixed-effects model was used. The unit of analysis was the individual vessel enhancement. The primary outcome was binary: If an infarction was in the territory of an inflamed vessel, this infarction was termed “matched” (match = 1, no match = 0). The analysis was restricted to assessments performed within a 50-day window surrounding the initiation of immunosuppressive therapy. This timeframe was chosen primarily to focus the analysis on the period of highest expected clinical effect.

The model included fixed effects for a cubic polynomial of time (days from immunosuppression) and for scaled imaging metrics of circularity, stenosis degree, lesion length, T1 TSE and T1 SPACE value. To account for non-independent observations, random intercepts for each patient and for each anatomical vessel location nested within the patient were specified.

## Results

The search yielded nine cases of PACNS from the University Hospital Bern and 14 from the Cantonal Hospital St. Gallen database, giving a total of 23 patients with a total of 204 MRI examinations.

### Demographical and clinical characteristics of the included patients

**Fig. 1:**
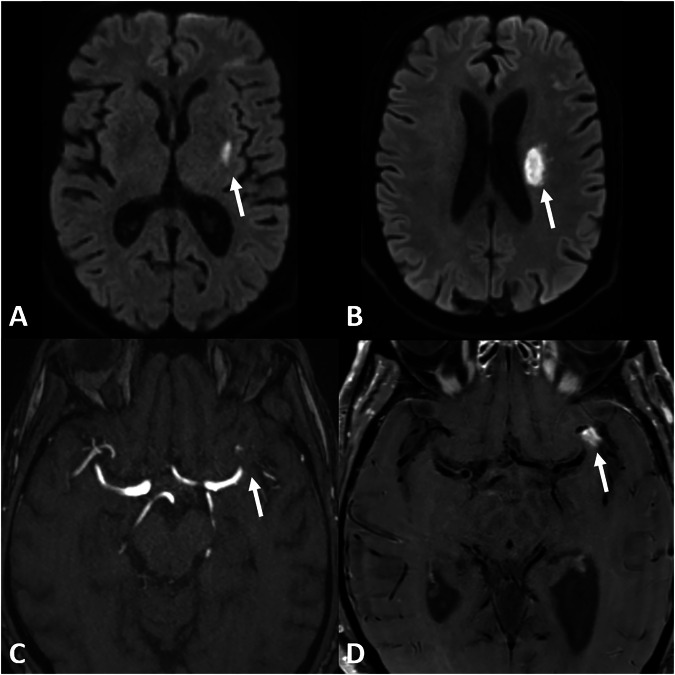
Initial imaging of a 74-year-old male patient who presented with right-sided hemiparesis and dysarthria. B1000 DWI (**A**, **B**) shows an infarction in the corona radiata left (arrow). There is a high-degree stenosis of the left MCA M1/M2 junctuion on TOF (arrow) (**C**) with focal vessel wall enhancement (arrow) (**D**) on T1 TSE dark blood. Therapy was initiated 1 day later with 1000 mg methylprednisolone for 3 days, followed by body weight-adapted prednisolone and cyclophosphamide

**Fig. 2:**
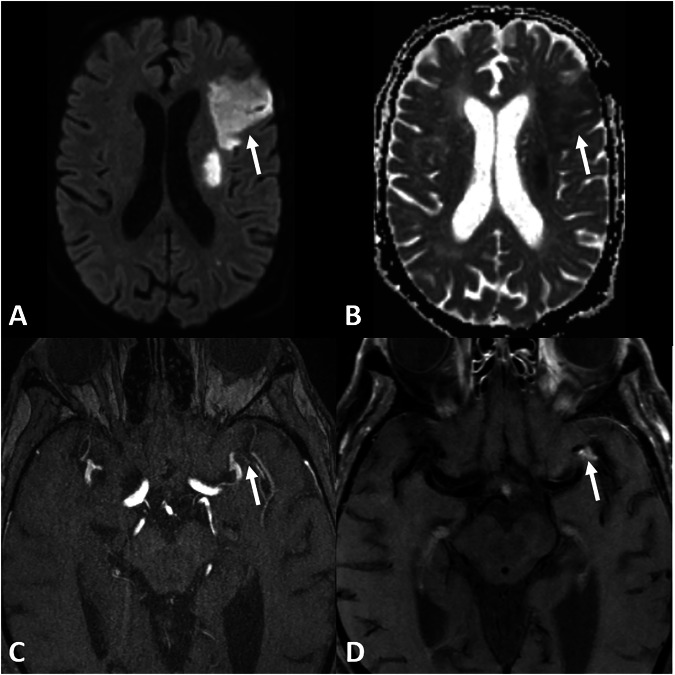
Follow-up imaging of the same 74-year-old male patient as Fig. 1, 13 days later, despite the initiation of immunosuppressant therapy. There is a new infarction on the DWI B1000 (arrow) (**A**) with decreased ADC values (arrow) (**B**) in the left MCA territory. The MCA stenosis remains constant (arrow) (**C**), whereas the vessel wall enhancement is slightly regressing (arrow) on T1 TSE dark blood (**D**)

**Fig. 3:**
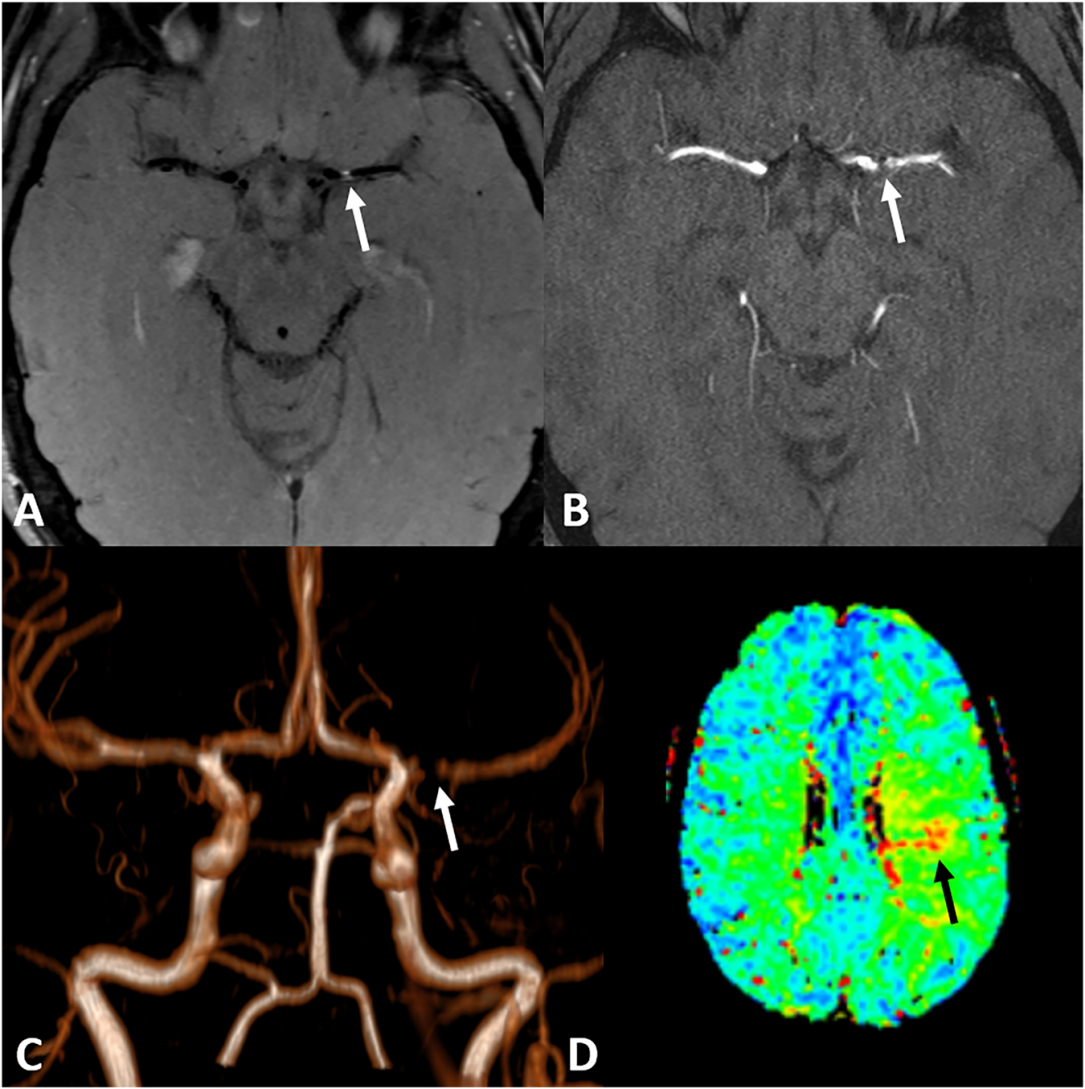
Initial imaging of a 29-year-old male patient, presenting with transient motoric aphasia. The proximal, left MCA shows focal vessel wall enhancement on axial T1 TSE dark blood (arrow) (**A**) with stenosis seen on TOF (**B**, **C**) (arrow) with reduced perfusion on time to peak (arrow) (**D**). No infarction was seen on DWI. Immunosuppressive therapy was subsequently initiated

**Fig. 4:**
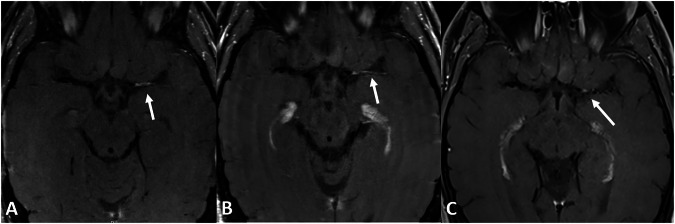
Follow-up imaging of the same 29-year-old male patient as Fig. 3 on axial T1 TSE dark blood patient shows vessel wall enhancement of the medial cerebral artery on the left side 1 month after therapy onset (**A**) that remains constant after 15 months (**B**) but is regressing after 4 years and 1 month (**C**) after treatment. No recurrent infarctions occurred; immunosuppressant therapy was stopped after 18 months

Mean age was 50 years (standard deviation ± 16.5 years), range 21–77 years. Fifteen out of 23 (65%) patients were male with a mean age of 52 years (± 16.0 years); the mean age of females was 46.1 years (± 18.7 years), differences were not statistically significant. Median time from symptom onset to therapy initiation was 0.4 months, range 0.1–26 months. DSA was performed in most cases (*n* = 13). Brain or meningeal biopsy was performed less frequently (*n* = 6). Demographics and individual treatments are given in Table 1. Imaging examples of initial and follow-up imaging in two patients are given in Figs. 1–4.

**Table 1:** Patient demographics and received treatments

	Cortisol pulse therapy	Cortisol induction therapy*	Induction therapy	Maintenance therapy	Follow-up
Male, 62 years	Methylprednisolone 500 mg/day, 4 days	Prednisolone 250 mg/day	Cyclophosphamide (every 4 weeks, 6 Cycles) total dose:8400 mg	Rituximab 1000 mg/6 months, only given once	Died 4 months after the start of maintenance therapy
Female, 40 years	Methylprednisolone 1000 mg/day, 3 days	-	Cyclophosphamide (every 4 weeks, 6 Cycles) total dose: 9000 mg. 10 times plasmapheresis	Rituximab 1000 mg/6 months and Cyclophosphamide 150 mg/day, switched to Mycophenolate 2000 mg/day	Complete remission, under therapy
Male, 36 years	Prednisolone 500 mg/day, 9 days	Prednisolone 1 mg/kg body weight/day	Cyclophosphamide (every 4 weeks, 6 Cycles) total dose:7200 mg	Azathioprine 150 mg/day	Complete remission, under therapy
Male, 54 years	Methylprednisolone 500 mg/day, 4 days*	Prednisolone 1 mg/kg body weight/day	Cyclophosphamide (every 4 weeks, 6 Cycles) total dose: 6000 mg.	Methotrexate 15 mg/week and Prednisolone 3.75 mg/day	Complete remission, under therapy
Female, 28 years	Methylprednisolone 100 mg/day, 5 days	Prednisolone 1 mg/kg body weight/day	Cyclophosphamide (every 4 weeks, 6 Cycles) total dose: 5100 mg.	Azathioprine 150 mg/day	Complete remission, under therapy
Female, 68 years	-	Prednisolone 1 mg/kg body weight/day	Cyclophosphamide (every 4 weeks, 6 Cycles) total dose: 3500 mg.	Azathioprine 150 mg/day	Complete remission, under therapy
Male, 61 years	Methylprednisolone 500 mg/day, 3 days	Prednisolone 1 mg/kg body weight/day	Cyclophosphamide (every 4 weeks, 6 Cycles) total dose: 10200 mg	Azathioprine 200 mg/day	Complete remission, after therapy
Male, 48 years	Methylprednisolone 2000 mg/day, 3 days	Prednisolone 1 mg/kg body weight/day	-	Azathioprine 100 mg/day, switched to Prednisolone 10 mg/day due to Exanthema	Complete remission, under therapy
Male, 50 years	Methylprednisolone 1000 mg/day, 3 days	Prednisolone 1 mg/kg body weight/day	Cyclophosphamide (every 4 weeks, 6 Cycles) total dose: 7020 mg	Azathioprine 100 mg/day	Complete remission, therapy stopped after 3 years
Female, 27 years	Methylprednisolone 1000 mg/day, 3 days	-	-	Prednisolone 15 mg/day	Complete remission, therapy stopped after 15 months
Male, 58 years	Methylprednisolone 1000 mg/day, 5 days	Prednisolone 1 mg/kg body weight/day	Cyclophosphamide (every 4 weeks, 6 Cycles) total dose: 6000 mg.	Azathioprine 100 mg/day	Complete remission, therapy stopped after 8 months
Male, 54 years	Methylprednisolone 1000 mg/day, 5 days	Prednisolone 1 mg/kg body weight/day	Cyclophosphamide (every 4 weeks, 6 Cycles) total dose: 9000 mg	Azathioprine 150 mg/day	Complete remission, under therapy
Female, 63 years	Methylprednisolone 1000 mg/day, 1 day	Prednisolone 1 mg/kg body weight/day	-	Azathioprine 100 mg/day	Complete remission, under therapy
Male, 63 years	Methylprednisolone 500 mg/day, 3 days*	Prednisolone 1 mg/kg body weight/day	Cyclophosphamide (every 4 weeks, 6 Cycles) total dose: 9000 mg	Azathioprine 150 mg/day	Partial remission, under therapy
Male, 56 years	Methylprednisolone 1000 mg/day, 3 days	Prednisolone 1 mg/kg body weight/day	Cyclophosphamide (every 4 weeks, 6 Cycles) total dose: 8400 mg.	Azathioprine 200 mg/day	Complete remission, under therapy
Male, 74 years	Methylprednisolone 1000 mg/day, 3 days	Prednisolone 1 mg/kg body weight/day	Cyclophosphamide (every 4 weeks, 6 Cycles) total dose: 6000 mg	Prednisolone 15 mg/day	Complete remission, under therapy
Male, 27 years	-**	Prednisolone 1 mg/kg body weight/day	Cyclophosphamide suspended	-	Complete remission, therapy stopped after 6 months
Female, 59 years	-**	Prednisolone 1 mg/kg body weight/day	-	-	Complete remission, therapy stopped after 6 months
Female, 62 years	Methylprednisolone 500 mg, 3 days*	Methylprednisolone 100 mg	Cyclophosphamide (every 4 weeks, 6 Cycles) total dose: 6000 mg.	Azathioprine 125 mg/day	Died 11 months after the start of maintenance therapy
Male, 29 years	Methylprednisolone 500 mg/day, 5 days*	Prednisolone 1 mg/kg body weight/day	-	-	Complete remission, therapy stopped after 18 months
Male, 77 years	Methylprednisolone 1000 mg/day, 5 days	Prednisolone 1 mg/kg body weight/day	-	-	Complete remission, therapy stopped after 6 months
Female, 21 years	Methylprednisolone 1000 mg/day, 5 days	Prednisolone 1 mg/kg body weight/day	-	-	Complete remission, therapy stopped after 13 months
Male, 26 years	Methylprednisolone 1000 mg/day, 4 days	Prednisolone 1 mg/kg body weight/day	-	-	Complete remission, therapy stopped after 14 months

In many cases, dose adjustments were necessary, for Azathioprin the final dose, that was used to achieve a clinically stable situation is given. For steroids, the initial dose is given, dose reductions frequently occurred

* Cortisol pulse was administered more than once due to delayed diagnosis

** No cortisol pulse was administered, since immunosuppressant therapy was started in a non-acute situation

### Description of the data sample

Each patient had a mean of 9.5 appointments ± 2.9 (SD). Mean time of follow-up MRI was 715 days, standard deviation ± 487 (SD): minimum 18 days, maximum 1496 days ± 3.6 (SD).

Raw TSE values for vessel wall enhancement were available for 22 patients, whereas raw SPACE values were only available for 15 patients. For 19 localizations across 14 patients, both raw TSE and SPACE values were reported. Measurements of the length of vessel wall enhancement, circular extent of enhancement and extent of stenosis were obtained in all 23 patients.

### Interobserver reliability

Agreement was good to excellent. ICC for different variables were: 0.969 (vessel wall signal intensity on TSE), 0.881 (vessel wall signal intensity on SPACE), 0.998 (length of enhancement) and 0.997 (stenosis). Cohen’s kappa was 1.0 (circular enhancement).

### Association between TSE and SPACE sequence measurements

The intra-reader association between TSE and SPACE scores (vessel wall enhancement and noise) was obtained from examinations with sequences available (*n* = 19). Observations were treated as independent. ICC values were low: Reader 1: ICC = 0.476, Reader 2: ICC = 0.406, Noise (Reader 1 only): ICC = 0.229. This low ICC led to the conclusion that TSE and SPACE values obtained for vessel wall enhancement are not comparable, and subsequently, vessel wall enhancement was investigated with standardized TSE metrics exclusively. A total of 371 unique vessel wall assessments were further evaluated.

### Prevalence of intracranial strokes after initiation of immunosuppressive therapy

Ischemic stroke after therapy onset occurred in 10/23 patients.

To calculate temporal distribution after therapy onset, we first had to evaluate whether or not infarctions occurred in the vascular territory of an affected vessel, e.g., an infarction of the middle cerebral artery territory could be caused by the middle cerebral artery inflammation, but also by inflammation of the ipsilateral internal carotid artery more downstream. If an infarction was in the territory of an inflamed vessel, this infarction was termed “matched.” A total of 76 distinct infarctions were documented across all vascular territories. Of these, 59 (77.6%) were matched to a corresponding vessel enhancement. Strokes not related to an inflamed vessel were not further evaluated due to uncertainty of the underlying pathology.

**Fig. 5:**
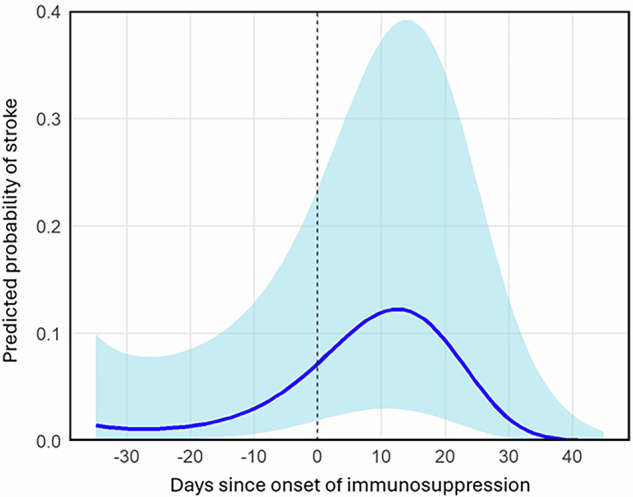
Predicted probability of ischemic strokes after initiation of immunosuppressive therapy. The figure depicts the results from the model calculation for stroke likelihood after initiation of immunosuppressive therapy. Time after therapy onset in days is given on the *x*-axis, and predicted probability is given on the *y*-axis. The highest probability of ischemic stroke is between 10 and 20 days after therapy onset, at approximately 12%

The temporal relationship between MRI assessment and immunosuppression initiation was significant. All three polynomial terms for time difference in days were statistically significant: the linear term (Estimate: −17.27, 95% CI: −31.81 to −2.74, *p* = 0.02), the quadratic term (Estimate: −17.12, 95% CI: −30.02 to −4.22, *p* = 0.01), and the cubic term (Estimate: −19.01, 95% CI: −32.11 to −5.92, *p* < 0.01). The complex nature of this temporal association is depicted by the estimated marginal means in Fig. 5, which illustrates the probability of a matched infarction relative to immunosuppression initiation.

### Inflamed vessel characteristics as predictors for strokes

**Fig. 6:**
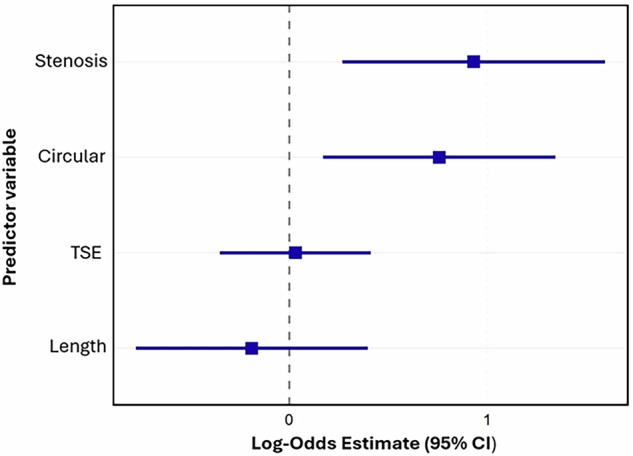
Forest plot of log-odds estimates from the GLMM. Stenosis and extent of circular enhancement are the strongest predictors for ischemic stroke after initiation of immunosuppressant therapy. Intensity of vessel wall enhancement on TSE sequences and the length of vessel wall enhancement do not predict strokes

Of the imaging features, a greater degree of stenosis (Estimate: 0.93, 95% CI: 0.27 to 1.59, *p* = 0.01) and higher vessel circularity (Estimate: 0.76, 95% CI: 0.17 to 1.34, *p* = 0.01) were significantly associated with a higher likelihood of a matched stroke. Lesion length (Estimate: −0.19, 95% CI: −0.77 to 0.40, *p* = 0.53) and scaled TSE value (Estimate: 0.03, 95% CI: −0.35 to 0.41, *p* = 0.87) were not significantly associated with strokes. These relationships are summarized in the forest plot in Fig. 6.

### Prevalence of intracranial hemorrhages after initiation of immunosuppressive therapy

Intracranial hemorrhage after the onset of therapy occurred in 5/23 patients. Most hemorrhages were small petechial hemorrhages (HI-1) without mass effect. In one case, a minor mass effect was visible (PI-1). Recurrent hemorrhage was visible in two patients and did not exceed HI-1. Due to the low number of observed hemorrhages, a more systematic analysis was not possible. Details are given in Table 2.

**Table 2:** Characteristics of intracranial hemorrhage after immunosuppressive therapy onset

Patient	Time since therapy onset	Severity of hemorrhage
Male, 62 years	23 days	HI-1
Male, 58 years	24 days and 244 days	HI-1
Male, 54 years	313 days	PI-1
Male, 74 years	7 days and 9 days	HI-1
Female, 62 years	5 days	HI-1

Severity of hemorrhage reflects the score for hemorrhagic transformation (HT), proposed by the European Cooperative Acute Stroke Study (ECASS) 2 [22]

## Discussion

This study evaluated the prevalence of ischemic stroke and intracranial hemorrhage in patients with primary angiitis of the central nervous system after initiation of immunosuppressant therapy and tried to associate vessel wall enhancement descriptors with risk scores of ischemic strokes.

The highest risk of ischemic stroke was between 10 and 20 days after initiation of immunosuppressant therapy, with a predicted probability of approximately 0.12 or 12%. The extent of stenosis and circular enhancement were predictors of stroke, whereas the intensity of vessel wall enhancement and length of vessel wall enhancement were not predictors of stroke. Intracranial hemorrhage was rare and deemed mostly insignificant.

The diagnosis of PACNS is difficult, and various criteria have been proposed. First described in 1988 by Calabrese and Mallek: (1) History of clinical findings of an acquired, otherwise unexplained neurologic deficit, (2) presence of classic angiographic or histopathologic features of angiitis within the CNS, and (3) no evidence of systemic vasculitis or of any other disorders that could cause or mimic the conventional angiography or pathologic features [4, 23]. Later, in 2009, Birnbaum and Hellman suggested revised criteria for the diagnosis of PACNS and added a level of certainty of the diagnosis, whereas in “definite,” as neuropathology confirmation is required, “probable” requires a high probability angiogram and abnormal findings on MRI and CSF profile [4, 13].

In this study, we intended to include only patients who most certainly had PACNS, and the aforementioned criteria were tightened. For us, the most important criterion was the decision of the interdisciplinary board, when long-term immunosuppressant treatment with potentially serious side effects indicated a reasonable amount of confidence.

Currently, MRI is probably the most important tool for the diagnosis of PACNS. Especially when stenoses in combination with vessel wall enhancement in relative absence of atherosclerosis are present. The recent European Stroke Organization (ESO) guideline on PACNS emphasizes the relevance of vessel wall imaging, but points out that the diagnostic work-up should be limited to expert centers. However, interpretation of a positive finding should not be the sole neuroimaging marker supporting the diagnosis of PACNS [4]. DSA still has its value and is especially useful in patients with clinical suspicion of PACNS when MR angiography is not diagnostic. The significance of ancillary findings such as leptomeningeal enhancement remains unclear, with minor impact on the final diagnosis [4].

Imaging differentials include atherosclerotic plaque, which may exhibit similar clinical symptoms, regardless of the severity of the stenosis, when intraplaque hemorrhage and inflammation occur [24], reversible cerebral vasoconstriction symptom (RCVS) [25] or moya-moya disease [26].

Manifestations of PACNS are often described as ischemic stroke or hemorrhage, but the ratio is not equal, as stroke is by far the most common symptom. Interestingly, 22.4% of strokes were observed without a corresponding vessel. The etiology of those strokes remains unclear. Comorbidities, such as makroangiopathy, are a possible explanation. Another explanation could be that the non-matched strokes are attributed to small vessel PACNS, which is invisible on MRI and can only be diagnosed by histopathology [4]. Therefore, in this study, we decided to choose and include only strokes with a clear etiology based on MRI to be as specific as possible.

### Limitations

The major limitations of the study were its multicenter and retrospective nature, with MR scans only performed as clinically indicated. Also, patient treatment did not follow a uniform protocol.

## Conclusion

Data obtained from this study may help radiologists and neurologists to identify patients at risk for stroke under immunosuppressant therapy. This may lead to a closer observation or even therapy adjustments in patients at risk. Overall, our results support the value of MRI-based vessel wall imaging as a reliable, non-invasive tool for risk stratification and disease monitoring in PACNS, potentially reducing reliance on more invasive procedures such as digital subtraction angiography.

## Supplementary information

ELECTRONIC SUPPLEMENTARY MATERIAL

## Data Availability

The dataset analyzed during this study is available from the corresponding author on reasonable request.

## Abbreviations

ADC: Apparent diffusion coefficient
CNS: Central nervous system
CSF: Cerebrospinal Fluid
DSA: Digital subtraction angiography
DWI: Diffusion weighted imaging
ECASS: European Cooperative Acute Stroke Study
HI: Hemorrhagic infarction
ICC: Intraclass correlation coefficient
LV: Large vessel
MRA: Magnetic resonance angiography
PACNS: Primary angiitis of the central nervous system
PH: Parenchymal hemorrhage
SI: Signal intensity
SPACE: Sampling perfection with application-optimized contrast using different flip-angle evolution
SV: Small vessel
TOF: Time of flight
TSE: Turbo-spin echo
