# Supplementary material for: Primary angiitis of the central nervous system: predictors of stroke during immunosuppressant treatment
Source: Insights Imaging. 2026 Feb 17;17:52. doi: 10.1186/s13244-026-02217-4 (PMC12913815; doi:10.1186/s13244-026-02217-4)
Supplement: Supplementary file 1 — ELECTRONIC SUPPLEMENTARY MATERIAL [file 13244_2026_2217_MOESM1_ESM.pdf]

Primary Angiitis of the Central Nervous System: Predictors of Stroke during immunosuppressant treatment

ELECTRONIC SUPPLEMENTARY MATERIAL

Supplementary Figure S1 Example of Measurements

Supplementary Figure S1. Example of Infarction and measurments in a 63 years old female patient, initial MRI prior to any treatment shows an infarction in the corona radiata, left side, MCA territory on DWI B1000 (A). Measurements of "enhancement length" of the affected left MCA on axial T1 dark blood after contrast was 15 mm (B) and "signal intensity" on axial T1 dark blood after contrast was 141 in this case (C) are shown. Measurment of "circumferential enhancment" (white arrow) of the affected left MCA on sagittal T1 dark blood SPACE was 270 degrees (D). "Stenosis" on axial TOF was 43% (E).

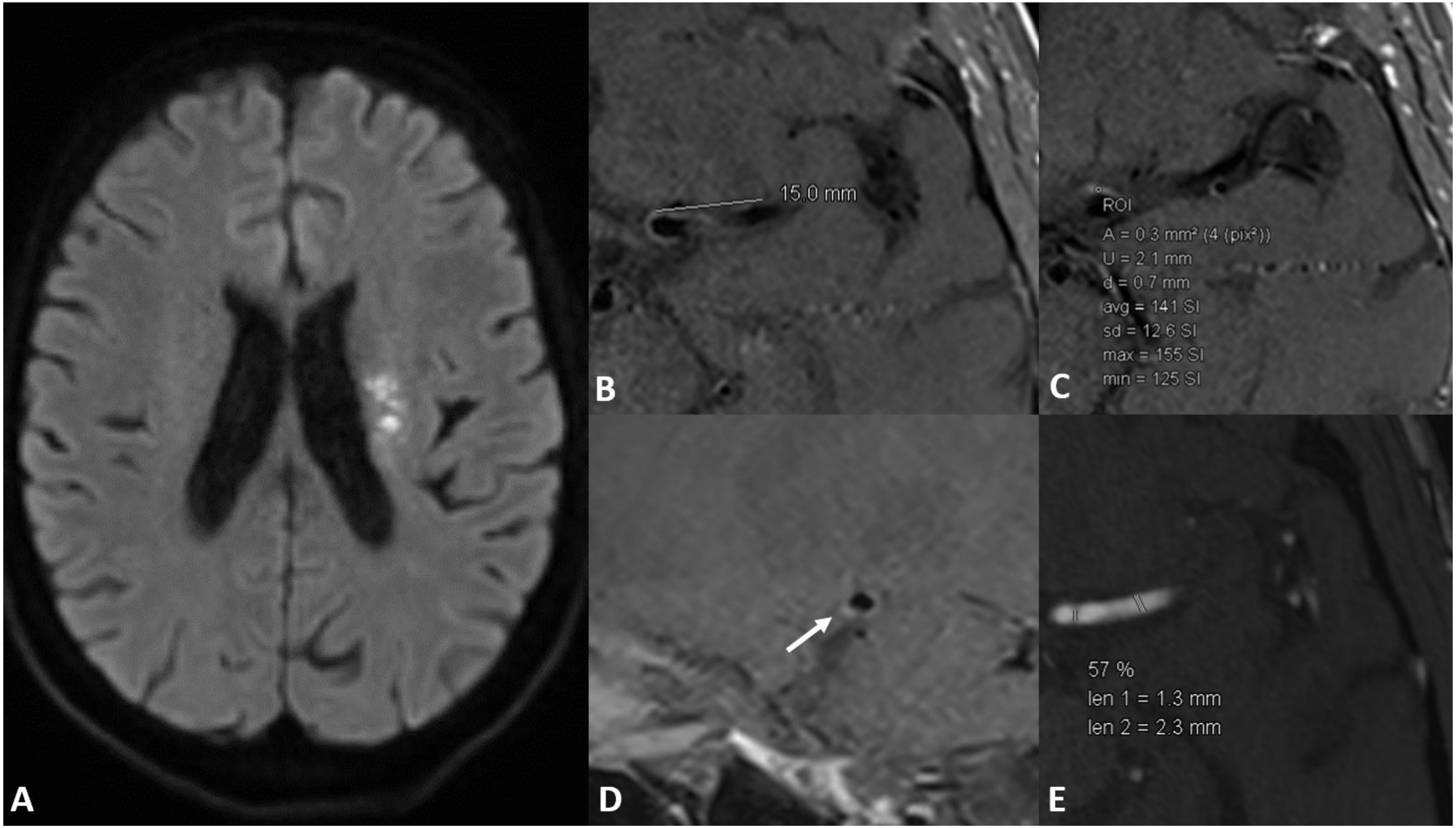
15.0 mm
ROI
A = 0.3 mm² (4 (pix²))
U = 2.1 mm
d = 0.7 mm
avg = 141 SI
sd = 12.6 SI
max = 155 SI
min = 125 SI
57 %
len 1 = 1.3 mm
len 2 = 2.3 mm
A
B
C
D
E
